# The Collective Direction of Attention Diffusion

**DOI:** 10.1038/srep34059

**Published:** 2016-09-28

**Authors:** Cheng-Jun Wang, Lingfei Wu, Jiang Zhang, Marco A. Janssen

**Affiliations:** 1 Computational Communication Collaboratory, School of Journalism and Communication, Nanjing University, Nanjing 210093, P.R. China; 2 Center for Behavior, Institutions and the Environment, Arizona State University, Tempe, AZ 85281, USA; 3 School of Systems Science, Beijing Normal University, Beijing 100875, P.R. China; 4 School of Sustainability, Arizona State University, Tempe, AZ 85281, USA

## Abstract

We find that the flow of attention on the Web forms a directed, tree-like structure implying the time-sensitive browsing behavior of users. Using the data of a news sharing website, we construct clickstream networks in which nodes are news stories and edges represent the consecutive clicks between two stories. To identify the flow direction of clickstreams, we define the “flow distance” of nodes (*L*_*i*_), which measures the average number of steps a random walker takes to reach the *i*th node. It is observed that *L*_*i*_ is related with the clicks (*C*_*i*_) to news stories and the age (*T*_*i*_) of stories. Putting these three variables together help us understand the rise and decay of news stories from a network perspective. We also find that the studied clickstream networks preserve a stable structure over time, leading to the scaling between users and clicks. The universal scaling behavior is confirmed by the 1,000 Web forums. We suggest that the tree-like, stable structure of clickstream networks reveals the time-sensitive preference of users in online browsing. To test our assumption, we discuss three models on individual browsing behavior, and compare the simulation results with empirical data.

In theory, information can have an infinite number of copies. This presents challenges for predicting the duration of information diffusion and the number of copies to be generated at each step. To address this problem, scholars investigate the competition for limited attention between information pieces^1^,^2^,^3^,^4^,^5^. A widely used model is called “clickstream networks”, in which nodes are information pieces and edges represent the successive clicks between nodes created by users. This model has been applied to uncover the hidden structure of semantic spaces^2^, create high-resolution human knowledge maps^1^, and analyze the diffusion of Internet memes^3^.

In previous studies on clickstream networks, a rarely mentioned topic is the direction of clickstreams. We assume that in an online system with many users, the randomness in the browsing behavior of individual users may cancel out with each other, giving rise to a system-level order that reveals the collective preference of users. Therefore, we should be able to identify the direction of clickstream diffusion at the collective level, which is an innovation compared to earlier literature. To verify our assumption, we analyze two datasets, the news story voting records from a news sharing website and the thread browsing records from 1,000 Chinese Web forums. We define “flow distance” *L*_*i*_, a network-based metric, to detect the direction of clickstreams. *L*_*i*_ measures the average number of steps a random walker takes to reach the *i*th node in clickstream networks. It is similar to “effective distance” proposed in ref. 6, but overcomes the latter’s limitation^7^ by considering all possible paths between two nodes and not just the shortest path. By sorting nodes in order of *L*_*i*_ we retrieve the global direction of clickstreams in the network from the complex local interactions between nodes; clickstreams are generally transported from nodes associated with low values of *L*_*i*_ to nodes who have high values of *L*_*i*_. Meanwhile, we find two variables characterizing the properties of nodes, the clicks to nodes *C*_*i*_ and the age of nodes *T*_*i*_, are related with *L*_*i*_. In the news sharing website, putting together these three variables gives a comprehensive understanding of the life cycle of news: as time goes by, the flow distance of news stories increases due to a lack of novelty and visibility, leading to the decline of clicks to these stories. The constant replacement of old news by the most recent news gives rise to a stable, tree-like structure of clickstream networks, in which the latest news stories are always located near the “root” and the earlier stories occupy the “leaf” positions. A systematic investigation of 1,000 Web forums not only confirms the tree structure of clickstream networks, but also predicts that, this structure is related with the scaling between users and clicks, which has been widely observed in online systems^5^,^8^. We suggest that the tree-like, stable structure of clickstream networks reveals the time-sensitive preference of users in online browsing. To test our assumption, we discuss three models on individual browsing behavior. In these models users generally visit webpages in reverse chronological order, but different models allow users to repeatedly visit pages in different ways. By comparing the mechanisms that allow users revisit the newest, the oldest, and the middle-age stories, respectively, we find that the model in which users revisit middle-age webpages presents the properties most similar to the real systems.

## Materials and Methods

### Data Sources

We analyze two datasets of web browsing activities, including DIGG and TIEBA. Digg (http://digg.com/) is a news sharing website. On this website, users submit new stories and vote for them. In particular, headlines of news are displayed on the homepage and sub-category pages (e.g., technology, entertainment, sports, etc.). To view a news story, a user clicks the headline and opens a new webpage that displays the full story. If the user likes the story, he gives a thumbs-up (also called “digg”) for that story. The stories will appear on the homepage if they obtain a critical mass of votes (diggs) quickly enough. Due to the limited space of the homepage, old stories are constantly replaced by new stories. The DIGG dataset under study includes 3 × 10^6^ votes to 3553 news stories created by 1.4 × 10^4^ users in a month^4^. Tieba (http://tieba.baidu.com/) is the largest Chinese web forum system managed by the Chinese search engine company, Baidu. On this platform, users can create new forums (“bar”) with custom, unique names. After a forum is created, users may start a new thread, post a reply in a particular thread, or click the headline of a thread to open a new page and read the content. Different from Digg, Tieba does not calculate the popularity of threads (news stories). Threads are displayed in reverse chronological order by the time of the latest reply. As a result, while most of threads are removed away from the homepage due to a lack of novelty, a few threads may sit on the homage for a long time. Out data set contains the thread browsing records on the top 1,000 forums created by more than 1 × 10^7^ users in 24 hours. Both DIGG and TIEBA are anonymous datasets and we do not have access to the personally identifiable information of users.

### Constructing Clickstream Networks

To obtain clickstream networks we split dataset into chunks of equal time span (a hour in TIEBA and a day in DIGG) and count the number of successive visits *w*_*ij*_ to two pages *i* and *j* within the given time period. For each *w*_*ij*_ we add a weighted, directed edge pointing from *i* to *j*. After all edges are included in the network, we apply a technique called “network balancing”. We add two artificial nodes “source” and “sink” and connect them to the existing nodes such that weighted in-degree (the sum of weights over inbound edges) equals weighted out-degree (the sum of weights over outbound edges) on each of the existing nodes^9^. These two artificially added nodes represent the “environment” of an online community, namely, other online communities and/or the offline world. Including the environment in the analysis allows us to investigate the complete clicking paths of users. All users come from the environment (“source”), enter into the system to click a sequence of webpages, and then leave from the system and back to the environment (“sink”). This information is particularly important if we want to analyze the transition probability of users between webpages. Note that besides the aforementioned method, there are also other methods for constructing the temporal paths^10^.

**Figure 1:**
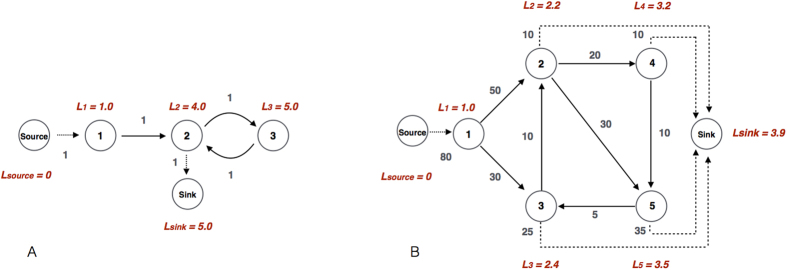
Two example clickstream networks in which nodes are webpages and edges represent the successive visits between webpages. Two artificial nodes, source and sink, are added to balance the network such that weighted in-degree (the sum of weights over inbound edges) equals weighted out-degree (the sum of weights over outbound edges) on each node except for source and sink themselves. Panel A shows that our method gives a finite *L*_*i*_ for nodes on loops. Panel B shows the conservation of clickstreams (Eq. 1 and Eq. 2) in the balanced networks. The number of leaving users is *D*_*i*_ = {0, 10, 25, 10, 35} from node 1 to node 6. The clicks on nodes, or the weighted in-degree (which equals weighted out-degree), are *C*_*i*_ = {80, 60, 35, 20, 40}. We find that 
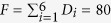
 and 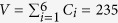, in which *F* and *V* represents the total number of users and clicks, respectively. For this network, we provide a video to show the iterative calculation of *L*_*i*_ available at https://www.youtube.com/watch? v=AxB-_OuCtlc.

Figure 1 presents two example clickstream networks, in which nodes are webpages and edges represent the successive clicks between webpages. Figure 1A gives a very simple network to illustrate that we can calculate finite flow distance for nodes on loops. In Fig. 1B we present a more complex network to illustrate the principle of clickstream conservation after networks are balanced. Clickstreams conservation means that (1) the total number of users entering into the system equals to the total number of users leaving the system; and (2) The total number of clicks created in the system equals the sum of clicks over all nodes. The clickstreams conservation can be expressed as


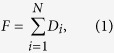


and


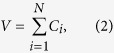


in which *N* is the number of nodes in the network. *F* and *V* represents the total number of users and clicks, respectively. *D*_*i*_ is the number of clicks transported to sink from the *i*th node. It also indicates the number of users leaving the system from the *i*th node. *C*_*i*_ is the total number of clicks generated on the *i*th node. It equals the weighted in-degree or the weighted out-degree of node *i*.

### Calculating Flow Distance *L*_*i*_

We define flow distance *L*_*i*_ as the average number of steps (edges) a user takes from source to reach the *i*th node. In Fig. 1 we show the flow distance of nodes using text in red. *L*_*i*_ can be calculated using either an iterative method or a matrix-based method. For the iterative calculation, we firstly set the initial values of *L*_*i*_ as one unit for all nodes. Starting for any arbitrarily selected node *i*, we calculate the average path length 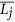 of all its upstream nodes *j*, weighted by the normalized form of *w*_*ji*_ (which is divided by the weighted in-degree of *i*). We then obtain 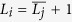 and continue to calculate the flow distance of the downstream nodes of *i*. The process goes on until the values of flow distance converge on all nodes. The iterative calculation is simple and fast in practice and is particularly powerful in handling large datasets. A similar method is used to calculate the PageRank values of webpages in large hyperlink networks^11^.

The iterative method is very useful in empirical data processing, but it does not give a formal definition of *L*_*i*_. Therefore, we derive the matrix-based definition of *L*_*i*_ based on a Markov model of clickstreams as follows. Firstly we obtain a weight matrix *F* from a clickstream network and normalize *F* by column to derive a new matrix *M*, whose element *M*_*ji*_ represents the probability that a user visiting node *i* comes from its upstream neighbor *j*. For the convenience of calculation, we transpose *M* to *M*^*T*^ such that 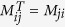, and let the sum of each row in *M*^*T*^ equals 1. We can write *L*_*i*_ as:


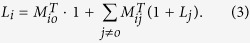


Eq. 3 holds because in order to reach node *i*, a user has to visit one of its upstream neighbors *j*, creating a path of length 1 + *L*_*j*_ with probability 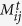. The only exception occurs when the random walker jumps to *i* directly from source, generating a path of length 1 with probability 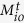. To solve Eq. 3 we use the condition that the sum of each row in *M*^*T*^ equals 1 and rewrite it as


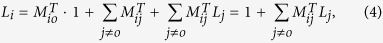


which tells us that, the flow distance from source to node *i* equals 1 plus the expected value of the flow distances from source to its upstream nodes *j*. Therefore, the previously discussed iterative calculation of *L*_*i*_ is proved to be correct. The matrix form of Eq. 4 reads


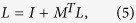


in which *I* is an all-ones vector. Eq. 5 can be solved as


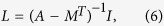


in which *A* is an identity matrix.

## Results

### The Life Cycle of News

In the age of information overload, news has to compete for the limited attention of users, i.e., attract clicks, in order to stay visible in the virtual world. This competition exists throughout the whole life cycle of news, leading to the constantly replacement of old news by the latest news^4^. In the analyzed Digg system, a news story has to obtain a critical mass of votes (diggs) in a short period (which is defined by the news ranking algorithm of Digg) in order to appear on the homepage. And sitting on the homepage will help it attract more votes quickly. But the increase of votes will gradually slow down and saturate due to the lack of novelty and visibility (when its position on the homepage is taken by the latest news). Besides the popularity ranking algorithm, other applications such as the interface displaying friends’ preferences may also influence users’ decision on popular stories to promote^12^. Therefore, the rise and decay of news is caused by the content switching mechanism of users, which reflects the preference of users moderated by the website algorithms designed to facilitate news voting.

**Figure 2:**
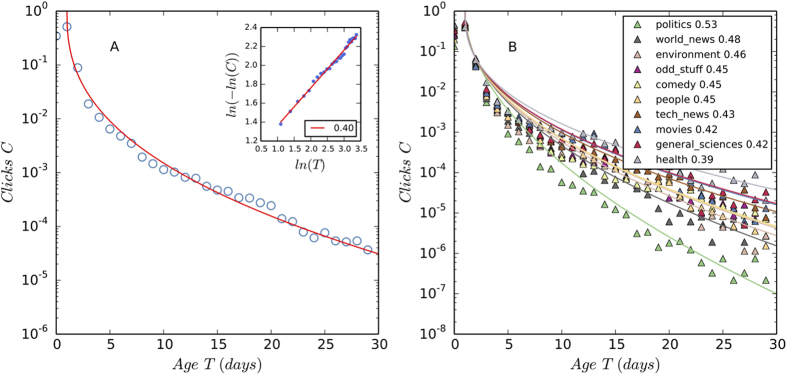
The temporal evolution of clicks. The daily clicks to news stories increase and reach a maximum value on the second day of news submission (T = 0 for the first day and T = 1 for the second day), and then decay over a relatively long time period. The decay of clicks is quantified by Eq. 7 with *ω* = 0.4. As shown in the inset of Panel A, we estimate the value of *ω* by fitting *ln*(−*ln*(*C*)) against *ln*(*T*) using OLS regression in a Log-log plot. Panel A shows that the theoretical prediction (the red curve) fits the empirical data (the blue circles) very well. In Panel B we fit the decay trends of the ten most voted news categories and find that the attention to politics news has the fastest decay, whereas the interest to health news lasts the longest.

We investigate the life cycle of news in DIGG and find that the daily clicks to news stories generally increase and reach a maximum value on the second day of news submission, and then decay over a relatively long time period. To quantify the decay period we write clicks *C* as a function of time *T* and find that *ln*(−*ln*(*C*)) scales to *ln*(*T*) linearly, or


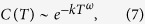


in which *k* and *ω* are constant parameters. Eq. 7 is called stretched exponential function or Kohlrausch-Williams-Watts function^4^. The parameter *ω* determines the decay rate of clicks. When *ω* > 0, clicks decrease slower than exponential function and faster than power law over time^4^. In Fig. 2A we estimate *ω* = 0.4 using the data of the ten most popular categories of news. Interestingly, while the decay of clicks generally follows Eq. 7, different categories of news have different decay rates. In particular, among the top ten categories, the attention to politics news has the fastest decay, and the interest to health news lasts the longest, as shown in Fig. 2B.

To better understand the temporal evolution of clicks to news, we construct clickstream networks to observe how users switch between news stories systematically and investigate whether the observed decay of attention is related with the change of the position of news in these networks. We trace the individual news voting streams of users on a daily basis and aggregate them to obtain daily networks in which nodes represent news stories and edges show the clickstreams between stories. As introduced in Materials and Methods, we add two artificial nodes, “source” and “sink” to represent the “environment” of clickstream networks, which could be other online communities and/or the offline world. Including the environment in the analysis allows us to investigate the complete clicking paths of users.

**Figure 3:**
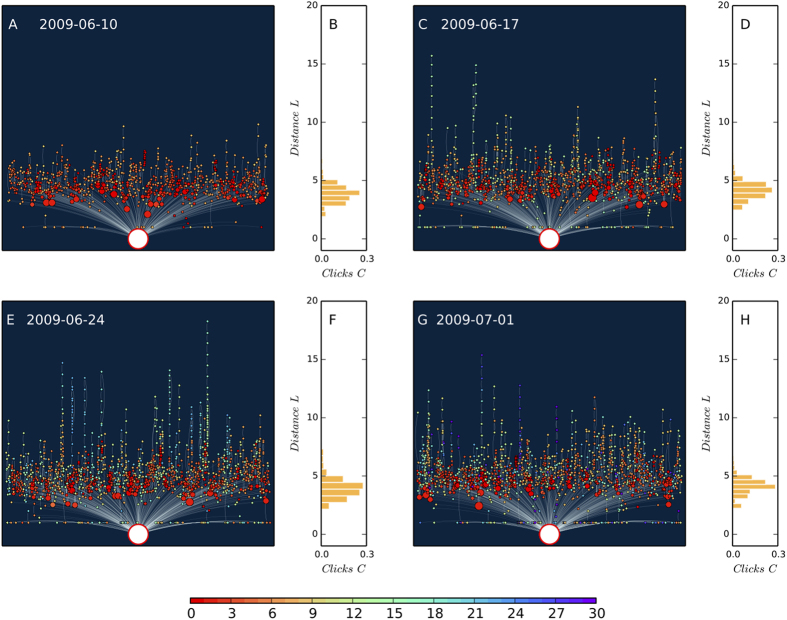
Four daily DIGG clickstream trees. The nodes are news stories and the edges are clickstreams. To generate these trees we select the maximum-weight inbound edge for nodes, i.e, each node is connected to the most important upstream neighbor. The size of nodes is proportional to their daily clicks and the colors show the age of nodes in days. The *y*-axis shows the flow distance *L*_*i*_ of nodes from source (the big white circle at the bottom of the trees) and the *x*-axis shows the horizontal values of nodes, which are calculated by the RT algorithm^19^ such that the edges do not overlap each other. The displayed networks share the same *y*-axis with the histograms on the right, which show the distribution of clicks on *L*_*i*_. It should be noted that although we use the maximum spanning tree to illustrate the direction of attention diffusion, the values of *L*_*i*_ are calculated in completed networks, thus the vertical order of nodes and the distribution of clicks on *L*_*i*_ are not affected by the tree-visualization method. By comparing the four daily networks we find that the distribution of clicks on flow distance seems to be invariant over time. Meanwhile, new stories (nodes of warm colors) are always closer to the source than old stories (nodes of cold colors), revealing the time-sensitive preference of news readers.

We find that the diffusion of clickstreams has a direction at the network level, revealing the collective preference of users in favor of new stories against old ones. In particular, we define a network-based metric, “flow distance” *L*_*i*_, to measure the expected number of steps (edges) a random walker takes from source to reach the *i*th story (see Materials and Methods for details). By sorting nodes in order of *L*_*i*_ we retrieve the global direction of clickstreams; they are generally transported from nodes associated with low values of *L*_*i*_ to nodes who have high values of *L*_*i*_. As shown by Fig. 3, the flow of clickstreams forms a directed, tree-like structure, and the distribution of clicks on *L*_*i*_ seems to be time-invariant, even though the positions of old nodes are constantly taken by new nodes.

**Figure 4:**
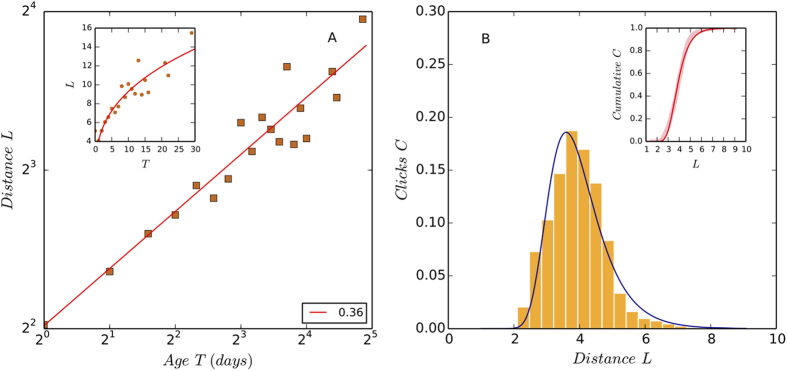
The relations between news age, flow distance, and the number of received clicks. Panel A shows that the flow distance of news stories scales to time sub-linearly. The data of different new stories has been binned to estimate the scaling exponent *ω*, which is 0.36 (p-value < 0.001, *R*^2^ > 0.8). To obtain a robust estimation we select news stories that has more than three data points and the Pearson correlation between age and distance of which is greater than 0.9. As shown by the inset of Panel A, the flow distance of news from source goes up rapidly at first and then the increasing speed becomes slower as time goes by. Panel B shows that the number of clicks (the orange bars) increases with the distance at first, and then decays with it gradually, forming a bell curve that can be quantified by the differential form of the Gompertz function (Eq. 10). The y-axis of the fitted curve is adjusted to be consistent with the histogram, whose hight depends on the number of bins. The turning point appears at the location *L* ≈ 4, which correspond to *T* ≈ 1 according to Fig. 4A. This observation is consistent with Fig. 2, which shows that the daily clicks to news increase and reach a maximum value on the second day of news submission (T = 0 for the first day of news submission and T = 1 for the second day), and then decay over a relatively long time. In data analysis we estimate the parameters *α*_1_ = 159.46 and *β*_1_ = 0.34 of the Gompertz function (Eq. 9) using the number of cumulative clicks (pink data points in the inset) and then plot the differential form (the blue curve).

It is observed that the flow distance of news scales to time sub-linearly, satisfying the function


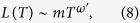


in which *m* and *ω*′ are constant parameters. In Fig. 4A we plot *ln*(*L*) against *ln*(*T*) in the log-log axes and estimate that *ω*′ = 0.35 using the OLS regression. The 95% Confidence Interval of *ω*′ is [0.25, 0.42]. According to the property of power functions, *ω* < 1 means that the flow distance of news goes up rapidly at first and then the increasing speed becomes slower as time goes by.

We find that the cumulative number of clicks *W* increases with flow distance monotonically, forming a S-shaped growth curve. While other functions are also available, we use the Gompertz function to model the growth curve. This is because the Gompertz function (and its derivative) is both simple in math and also widely used to characterize the growth dynamics of complex systems^13^.


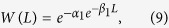


in which *α*_1_ and *β*_1_ are constant parameters. *α*_1_ determines the horizontal position of the midpoint of the function (where *W*(*L*) = 0.5) and *β*_1_ affects how steeply the function rises as it passes through its midpoint. The shape of *W*(*L*) characterizes the clickstream production behavior of the system. Increasing the value of *α*_1_ will move the curve to the right, increasing the fraction of users of long surfing paths. These two parameters are estimated to be *α*_1_ = 159.46 and *β*_1_ = 0.34 in Fig. 4B.

From Eq. 9 we know that the function of clicks on flow distance should be the differential equation of the Gompertz function, that is,


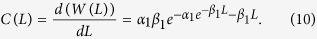


As shown by Fig. 4B, the number of clicks increases with flow distance at first and then decreases with it. The turning point appears at the location *L* ≈ 4, which corresponds to *T* ≈ 1 according to Fig. 4A. This observation is consistent with Fig. 2, which shows that the daily clicks to news increase and reach a maximum value on the second day of news submission (T = 0 for the first day and T = 1 for the second day in Fig. 2), and then decay over a relatively long time period. As we focus on the decay trend of clicks, we only need to analyze the behavior of Eq. 9 and Eq. 10 when *T *≥ 1 and *L* ≥ 4. Considering the empirical values of *α*_1_ and *β*_1_, we know that when *L* ≥ 4, the first term in the exponent of Eq. 10 is approximately 0, giving


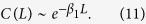


Putting together Eq. 8 and Eq. 11 leads to


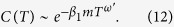


Comparing Eq. 12 with Eq. 7, we predict that *ω*′ ≈ *ω*. In empirical data analysis, we find that the value *ω* = 0.4 lies in the 95% Confidence Interval of *ω*′, that is, [0.25, 0.42]. This means that the studied clickstream networks preserve a stable daily structure that allows the prediction of clicks to news stories by their positions in networks.

### The Scaling of Clickstream Networks

In the last section we constructed clickstream network and analyzed the relationship between three variables of news stories, including age (*T*), the distance from source (*L*), and clicks (*C*). We find that due to the existence of a stable clickstream structure, we can predict the clicks to news stories from their position in clickstream networks. To further investigate the observed stable structure of clickstream networks, we will analyze the TIEBA dataset, which includes the clicking records generated by more than 1 × 10^7^ users in 24 hours on 1,000 web forums. If the clickstream networks always preserve the similar structure over time, we should be able to obtain a robust relationship between users and clicks, as discussed in^5^,^8^,^14^. Our analysis is presented as follows.

We have shown that the cumulative number of clicks *W*(*L*) increases with flow distance *L*, forming a S-shaped curve that can be fitted by the Gompertz function, as described by Eq. 2 and Fig. 4B. We define another quantity *U*(*L*), that is, the fraction of users leaving the system from nodes of a flow distance smaller than *L*. We find that *U*(*L*) also has an S-shape, i.e., we have:


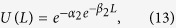


Putting Eq. 9 and Eq. 13 together, we achieve a more comprehensive understanding on the metabolism of clickstreams in online systems. We fit these two equations using the clickstream networks collected from the EXO (which is the name of a Korean band) forum and find that the values of *β*_1_/*β*_2_ and *α*_1_/*α*_2_ do not fluctuate a lot over time, supporting our assumption on the time-invariant structure of clickstream networks. In particular, the mean of *β*_1_/*β*_2_ is 0.96 and the mean of *α*_1_/*α*_2_ is 1.17. The standard deviations (SD) of both variables are 0.08, which are very small compared to the means.

**Figure 5:**
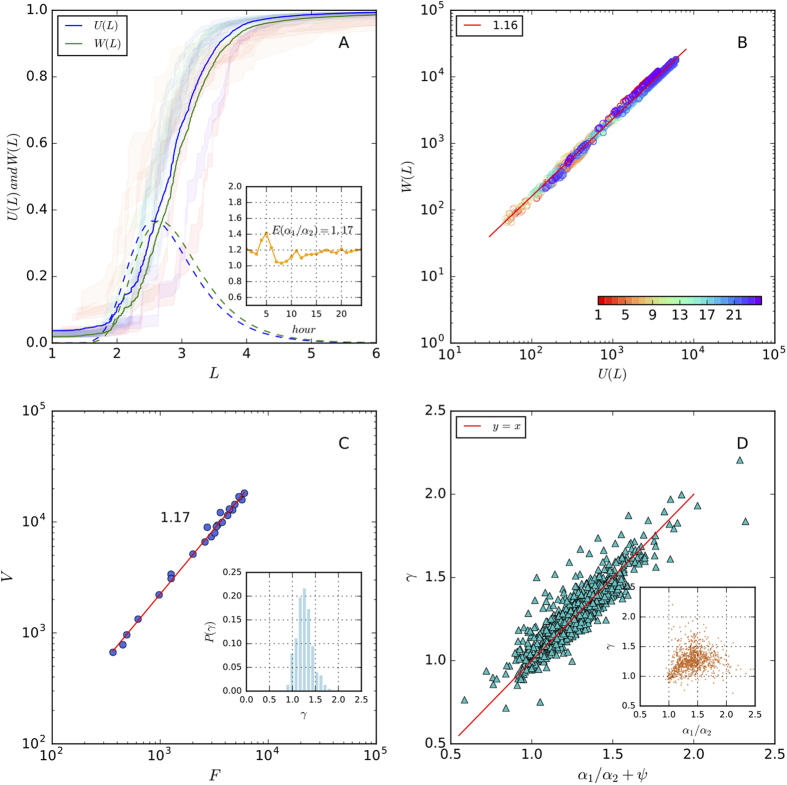
The scaling of clickstream networks. Panel A shows the increase of the cumulative number of generated clicks (the right bounds of the colorful bands) and leaving users (the left bounds) within distance *L* in 24 hours of the EXO forum. The color scheme for data in different hours is shown in the inset of Panel B. We also calculate the average values of *U*(*L*) (the blue curve) and *W*(*L*) (the green curve) and their differential forms (the dotted curves) over 24 hours. The inset of Panel A display the fluctuation of *α*_1_/*α*_2_ over hours. The mean value is 1.17 and the standard deviation is 0.08, which is small compared to the mean value. Panel B shows the scaling between *W*(*L*) and *U*(*L*) in each hour. The average scaling exponent is 1.16, which is very close to the average value of *α*_1_/*α*_2_. Panel C shows the scaling between users and clicks across hours, the value of the scaling exponent is 1.17, which is equal to the average value of *α*_1_/*α*_2_, too. In Panel D we test Eq. 20 using the data of the top 1,000 TIEBA forums and find that the theoretical prediction is supported by data. Each data point in Panel D represents a Web forum, and the distribution of the empirically estimated values of *γ* is shown in Panel C.

In Fig. 5A we show the relationship between *U*(*L*) (the upper bound of bands) and *W*(*L*) (the lower bound of bands) in each hour. Generally, these two curves have the similar S-shape, but are separated by a gap between them. As *α* in the Gompertz function determines the location of curve on the horizontal axis, the value of *α*_1_/*α*_2_ controls the width of the gap. In particular, *α*_1_ determines the mode value of surfing lengths at the individual level (see the dashed blue curve in Fig. 5A for example) and *α*_2_ determines the mode value of the surfing lengths aggregated at the webpage level (see the dashed green curve in Fig. 5A for example). If we fix the value of *α*_1_ and compare two online systems *A* and *B*, in which *α*_2_ is greater in *B* than in *A*, we can infer that while a majority of users have a similar surfing length in two systems, there are more users who have long surfing paths in system *B*, increasing the mode value of surfing length at the webpage level. In other words, while most users leave system B within a few steps, a few users visit a lot of threads, generating long surfing paths. Therefore, the gap between *U*(*L*) and *W*(*L*) reflects the inequality of click contribution among users. And the larger the gap is, the more unequal the click contributions are^15^,^16^,^17^.

Using the condition that *β*_1_/*β*_2_ ≈ 1 and *α*_1_/*α*_2_ > 1, we combine Eq. 9 and Eq. 13 and derive


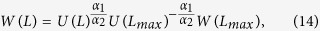


Note that when *L* reaches the maximum value, *W*(*L*) and *U*(*L*) equals the total number of clicks and users in the network. Considering Eq. 1 and Eq. 2, we have


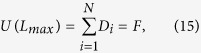


and


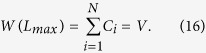


Therefore, Eq. 14 reads


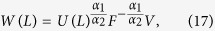


which predicts that within each network, *W*(*L*) always scales to *U*(*L*) super-linearly. This prediction is supported by Fig. 5B. Meanwhile, we find that equation


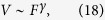


also holds (see Fig. 5C). Putting Eq. 17 and Eq. 18 together we have


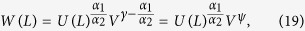


or,


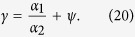


If we treat *ψ* as random noise, Eq. 20 allows us to predict *γ* from *α*_1_/*α*_2_. This is non-trivial because the value of *α*_1_/*α*_2_ can be obtained by analyzing a single, randomly selected hourly network, whereas *γ* can only be obtain through a collection of networks over many hours. To verify our assumption, we systematically investigate the 1,000 forums in the TIEBA dataset and find that Eq. 20 is supported by the empirical data (Fig. 5D).

To summarize, our analysis shows that directed, tree-like structure of clickstream networks, which remains stable over time, leads to the scaling between user and clicks. In particular, the gap between the increase of clicks and users with flow distance, which reflects the inequality of click contribution among users, determines the super-linear scaling *γ* of the total number of clicks against user population.

## Conclusions and Discussions

The previous empirical analysis in this paper reveals the time-sensitive nature of news and thread browsing activities at the collective level. However, the content switching mechanism at the individual level still remains unclear. Proposing a universal mechanism for individual browsing goes beyond the scope of the current paper, but we would like to present some preliminary results towards this direction to inspire further studies. We assume that users visit webpages in reverse chronological order, but in different models users return to previously visited nodes following different rules. In particular, users revisit the newest, the oldest, and the middle-age webpages in three models, respectively. We compare three models on individual browsing behavior.

**Figure 6:**
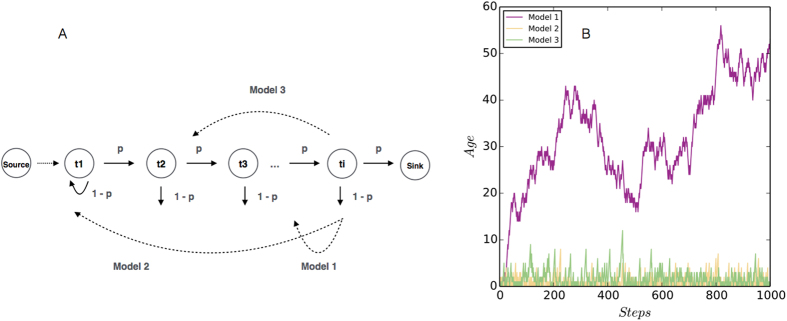
Panel A shows the schematic representations of three Web browsing models. The models have only two parameters, the way-back searching probability *p* and the number of clicks *N*. The indexed nodes show the webpages sorted by age, i.e., node *t*_1_ represents the most recently created webpage and node *t*_*i*_ represent the webpage that exists in the system for *i* time steps. For all the three models we assume that a typical user starts browsing by visiting the newest page and then continues by clicking older pages. For each step, we assume the probability of visiting the next page is *p*, and the probability of going back to previously visited pages is 1 − *p*. Model 1 assumes that the user only goes back to the last visited page *t*_*i*−1_. Model 2 assumes that the user goes back to *t*1. Model 3 assumes that the webpage in the mid-range between *t*_1_ and *t*_*i*_, i.e., the *i*/2th page, is selected. In Panel B we compared the simulation results for *p* = 0.5 and *N* = 1000 between three models. We find that Model 1, also called “bounded random walk model”^18^, allows users to explore very old pages, whereas in Model 2 and 3 only the newest pages are “visible”. Therefore, Model 2 and 3 give time-sensitive dynamics that is more similar to the empirical results.

**Figure 7:**
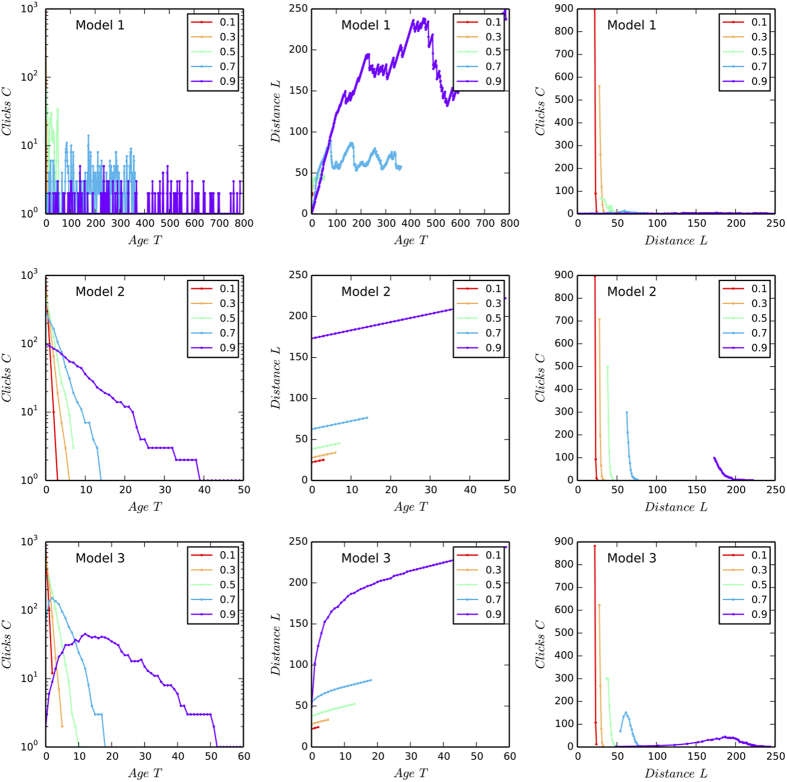
The dynamics of three Web browsing models. For each of the three models we set *N* = 1000 and change the values of *p* to observe the relationships between the age *T*, clicks *C*, and flow distance *L* of nodes. It is observed that Model 3 presents more similar dynamics to empirical findings than the other two models. In particular, when *p* *>* 0.5 in model 3, the flow distance *L* increases sub-linearly with the age of webpages *T*, and the distribution of clicks on *L* is unimodal. These are all the properties of empirical systems, which the other two models failed to present.

Panel A in Fig. 6 gives the schematic representations of the three models. All the three models have two parameters, the way-back searching probability *p* and the number of clicks *N*. The indexed nodes show the webpages sorted in reverse chronological order. In particular, node *t*_1_ represents the newest webpage and node *t*_*i*_ represent the webpage that was added into the system *i* time steps ago. We assume that a typical user starts browsing by visiting *t*_1_ and then continues by clicking earlier nodes. For each step of browsing, we assume the probability of visiting the next page is *p*, and the probability of returning back to previously visited pages is 1 − *p*. Meanwhile, starting from *t*_1_, a user either continue to visit *t*_2_ with probability *p* or return to *t*_1_ again with probability 1 − *p*. As for the returning rules, Model 1 only allows one-step return from *t*_*i*_ to *t*_*i* −1_. Model 2 assumes that users always return from *t*_*i*_ to *t*_1_. Model 3 assumes that users return from *t*_*i*_ to the webpage in the mid-range between *t*_1_ and *t*_*i*_, i.e., the *i*/2th page. In Fig. 6B we compared the simulation results for *p* = 0.5 and *N* = 1000 between three models. We find that Model 1, also called “bounded random walk model”^18^, allows users to explore very old webpages, whereas Model 2 and 3 only allow users to explore the newest pages. Therefore, Model 2 and 3 give time-sensitive dynamics that is more similar to that in real systems. In Fig. 7 we systematically compare the relationships between clicks *C*, age *T*, and flow distance *L* across three models. It is observed that Model 3 presents more similar dynamics to empirical findings than the other two models.

In summary, we analyze the directed, tree-like structure of attention diffusion. We develop a network-based metric flow distance *L* to detect the direction of attention flow and suggest that, by putting together *L*, clicks *C*, and age *T* we are able to quantify the competition for attention throughout the entire life cycles of news, which are relevant to their positions in the clickstream networks. The structure of clickstream networks is found to be stable over time, leading to the scaling between users and clicks. Finally, we compare three time-sensitive individual browsing models and find that the model in which users revisit the middle-age webpages gives rise to the dynamics that is most similar to real systems.

## Additional Information

**How to cite this article**: Wang, C.-J. *et al*. The Collective Direction of Attention Diffusion. *Sci. Rep.* **6**, 34059; doi: 10.1038/srep34059 (2016).
